# Proteomics for the Investigation of Surface-Exposed Proteins in Probiotics

**DOI:** 10.3389/fnut.2019.00052

**Published:** 2019-04-24

**Authors:** Rosa Anna Siciliano, Rosa Lippolis, Maria Fiorella Mazzeo

**Affiliations:** ^1^Institute of Food Sciences, National Research Council (CNR-ISA), Avellino, Italy; ^2^Institute of Biomembranes, Bioenergetics and Molecular Biotechnologies, National Research Council (CNR-IBIOM), Bari, Italy

**Keywords:** proteomics, probiotics, proteosurfaceome, surface-exposed proteins, S-layer proteins, moonlighting proteins, immunomodulation

## Abstract

Probiotics are commensal microorganisms that are present in the intestinal tract and in many fermented foods and positively affect human health, promoting digestion and uptake of dietary nutrients, strengthening intestinal barrier function, modulating immune response, and enhancing antagonism toward pathogens. The proteosurfaceome, i.e., the complex set of proteins present on the bacterial surface, is directly involved as leading actor in the dynamic communication between bacteria and host. In the last decade, the biological relevance of surface-exposed proteins prompted research activities exploiting the potentiality of proteomics to define the complex network of proteins that are involved in the molecular mechanisms at the basis of the adaptation to gastrointestinal environment and the probiotic effects. These studies also took advantages of the recent technological improvements in proteomics, mass spectrometry and bioinformatics that triggered the development of *ad hoc* designed innovative strategies to characterize the bacterial proteosurfaceome. This mini-review is aimed at describing the key role of proteomics in depicting the cell wall protein architecture and the involvement of surface-exposed proteins in the intimate and dynamic molecular dialogue between probiotics and intestinal epithelial and immune cells.

## Introduction

Probiotics are commensal microorganisms that are present in the intestinal tract and in many fermented foods, and are defined as “live microorganisms that, when administered in adequate amounts, confer a health benefit on the host” (1). Most probiotics are Gram-positive bacteria, mainly lactic acid bacteria (LAB), and Bifidobacteria (2). More recently, *Propionibacterium freudenreichii*, a beneficial bacterium traditionally used as a cheese ripening starter, has been recognized to exhibit probiotic abilities, some of these due to the production of nutraceuticals and beneficial metabolites (3). The mechanisms by which probiotics positively affect human health include promotion of digestion and uptake of dietary nutrients, strengthening of intestinal barrier function, modulation of immune response and enhancement of antagonism toward pathogens, either by producing antimicrobial compounds or through competition for mucosal binding sites (4, 5).

Surface-exposed proteins constitute the first-line of contact between bacteria and host being leading actors in this complex interplay as they directly interact with epithelial and immune cells (6). This interaction could lead to the inhibition or activation of key signaling pathways in the intestinal cells involving nuclear factor-κB (NFκB) and mitogen-activated protein kinases (MAPKs), and influencing the regulation of downstream pathways such as the secretion of cytokines (chemokines and interleukins) responsible for the immunomodulation or antibacterial peptides (defensins). The activation of these cascades prompts physiological modifications (increase of mucin secretion, changes in the surface properties, rearrangement of the tight junctions, etc.) and changes in genetic expression that could affect cell proliferation and survival (7–9).

The in-depth analysis of the proteosurfaceome, defined as “the proteinaceous subset of the surfaceome found at the cell wall and totally or partially exposed on the external side of the cell membrane,” represents a crucial point to elucidate molecular mechanisms underlying host/probiotic crosstalk (10, 11). In general, sorting of bacterial proteins to the cell surface is governed by the presence or absence of signal peptides which direct them to the protein export machinery thus allowing their migration to bacterial surface, and surface-retention domains responsible for their anchoring to cell wall or cytoplasmic membrane (12). Surface proteins can be mainly divided in four groups: (i) proteins anchored to the cytoplasmic membrane by hydrophobic transmembrane domain(s) (integral membrane proteins, IMP), (ii) lipoproteins which are covalently attached to membrane lipids after cleavage of a signal peptide by signal peptidase II, (iii) proteins containing C-terminal LPXTG-like motif and covalently attached to peptidoglycan by sortases, and (iv) non-covalently bound proteins which are associated to the cell wall through weak interactions (van der Waals forces, hydrogen or ion bonds) taking advantage of conserved structural domains (LysM proteins, WXL proteins, GW proteins, proteins with choline binding domains). Some bacteria can also show supramolecular structures, formed by the assembly of specific protein subunits on the cell envelope, such as flagella, mainly involved in cell motility, and pili or fimbriae, mainly involved in cell adhesion, aggregation, and immunomodulation (10, 11, 13). Furthermore, in several bacterial species (*Lactobacillus acidophilus, Lactobacillus helveticus, P. freudenreichii*, etc.) the outermost constituent of the cell wall is represented by a S-layer that is a semiporous proteinaceous crystalline array composed of self-assembling (glyco)protein subunits called S-layer proteins (SLPs) which act as a scaffold for non-covalently attached secreted proteins (also named S-layer associated proteins, SLAPs). SLPs are involved in several processes including maintaining cell shape, acting as molecular sieves, serving as binding sites, protecting against environmental stresses and mediating bacterial adhesion and gut immune response (14, 15). Noteworthy, cytoplasmic housekeeping proteins (metabolic enzymes, molecular chaperones, translational elongation factors, ribosomal proteins, etc.) have been identified in bacterial surface proteomes. Such proteins, defined as anchorless proteins or moonlighting proteins, display different, seemingly unrelated, functions in different cell locations, and lack any extra-cytoplasmic sorting sequence or binding domain, so that non-canonical secretion pathways have been hypothesized (16, 17).

Subcellular localization of bacterial proteins has been postulated by several bioinformatics tools mainly based on predictive algorithms. The most widely used tools are PSORTb v3.0 server that allows to obtain subcellular localization prediction on the basis of a multi-component approach including different modules such as SCL-BLAST for homology-based prediction, HMMTOP transmembrane helix prediction tool and a signal peptide identification tool (18, 19) and SignalP server, that predicts the presence and location of signal peptide cleavage sites in amino acid sequences from prokaryotes and eukaryotes (20). Prediction of transmembrane-spanning domains (helices) in IMP could be carried out by TMHMM v2.0 (21). Prediction of non-classically secreted proteins (such as moonlighting proteins) could be achieved by SecretomeP server (22) and MoonProt (23).

In the last decades, proteomics significantly contributed to depict an overall picture of the proteosurfeoceome through the identification of hundreds of proteins in a single analysis. In fact, this approach provided direct information on protein localization and topology, thus corroborating the bioinformatics prediction with experimental evidence, and allowed the identification of moonlighthing proteins, “unexpectedly” present on cell surface. More importantly, as the proteosurfaceome is a highly dynamic entity, tightly modulated by the host/bacteria molecular dialogue, proteomics represent the most suitable tool to monitor the wide reorganization of the surface proteins induced by either gastro intestinal tract (GIT) environment or other growth conditions, that could modulate the probiotic functionalities.

**Figure 1:**
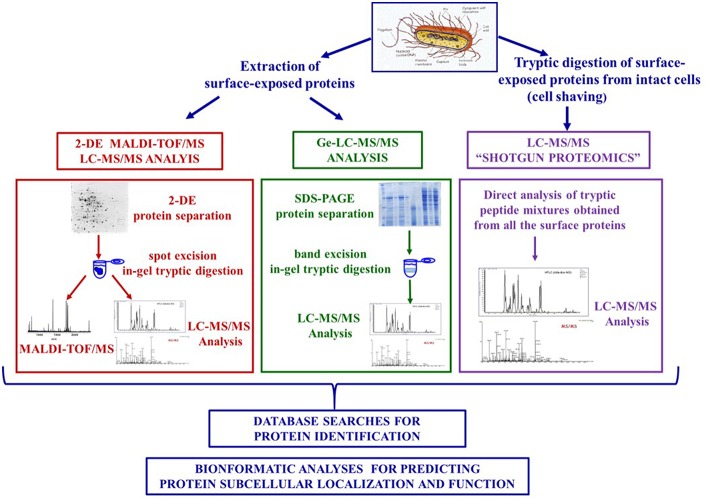
Proteomic strategies generally applied in the analysis of surface-exposed proteins.

However, the proteomic analysis of these proteins proved to be a challenging task due to their low abundance and hydrophobicity. First generation proteomic strategies integrated protein extraction from subcellular fractions, two dimensional electrophoresis (2-DE) and mass spectrometry, i.e., Matrix Assisted Laser Desorption Ionization—Time of Flight Mass Spectrometry (MALDI-TOF-MS) or nano-liquid chromatography coupled to tandem mass spectrometry (LC-MS/MS) and usually led to the identification of a limited number of surface proteins as these are hardly amenable to 2-DE analysis. The need to overcome these experimental drawbacks successively prompted the design of sophisticated strategies that selectively targeted surface proteins either by proteases digestion (shaving procedures) or biotin labeling (protein biotinylation procedures) of intact cells (24). These approaches exploited technological advancements in mass spectrometry and proteomics that allowed the development of gel-free proteomic strategies (also named shotgun proteomics) based on the direct analysis of peptides obtained from the tryptic digestion of the entire proteome by LC-MS/MS and Ge-LC-MS/MS approaches that include a preliminary SDS-PAGE fractionation step of the extracted proteins (25) (Figure 1).

This mini-review is aimed at describing both methods applied in the last years to define the bacterial proteosurfaceome and proteomic studies that significantly contributed in depicting the cell wall protein architecture and the involvement of surface-exposed proteins in the intimate and dynamic molecular dialogue between probiotics and host.

## Proteomic Methods for the Analyis of Surface-Exposed Proteins

### Protocols for Surface Protein Extraction

The analysis of surface proteins of Gram positive bacteria takes advantage of the cell envelope structure consisting of a cross-linked peptidoglycan layer. Cell wall degradation of intact cells is carried out for a short time (30–60 min) using enzymes such as lysozyme, mutanolysin (glycosidases), and/or lysostaphin (endopeptidase) in a buffer containing protease inhibitors and high sugar concentrations to create an osmotic pressure in order to maintain protoplast integrity and prevent contamination of cytoplasmic proteins. Surface proteins contained in the supernatants are recovered by centrifugation, precipitated, separated by 2-DE, and identified by mass spectrometry (26–29) (Figure 1). Denaturating agents (urea, guanidine-HCl, SDS buffers) have been also used to extract non-covalently bound surface proteins. Different protocols tailored to specific bacterial features have been proposed to ameliorate protein recovery and mass spectrometric analyses (26, 30, 31).

Surface proteins from S-layer forming bacteria are usually extracted using 5 M lithium chloride (LiCl) solutions thus breaking the hydrogen bonds that stabilize these supramolecular structures (32). More recently, a modified LiCl extraction protocol has been set up by Johnson et al. (33) to specifically separate the highly abundant SLPs from the less abundant SLAPs, exploiting their different solubility in 1 M LiCl solution, and applied to study SLAPs isolated from different probiotic bacteria (34–36).

As to Gram negative bacteria, classical methods for the extraction of surface exposed proteins take into account the peculiar features of their cell-envelope and include the subfractionation of outer membrane, cytoplasmic membrane and periplasmic proteins. Commonly, inner and outer membrane vesicles are prepared by lysozyme-EDTA lysis or French press lysis of bacteria, separated by density centrifugation using a sucrose gradient and analyzed by proteomics (37, 38).

### Cell Shaving Strategy

**Figure 2:**
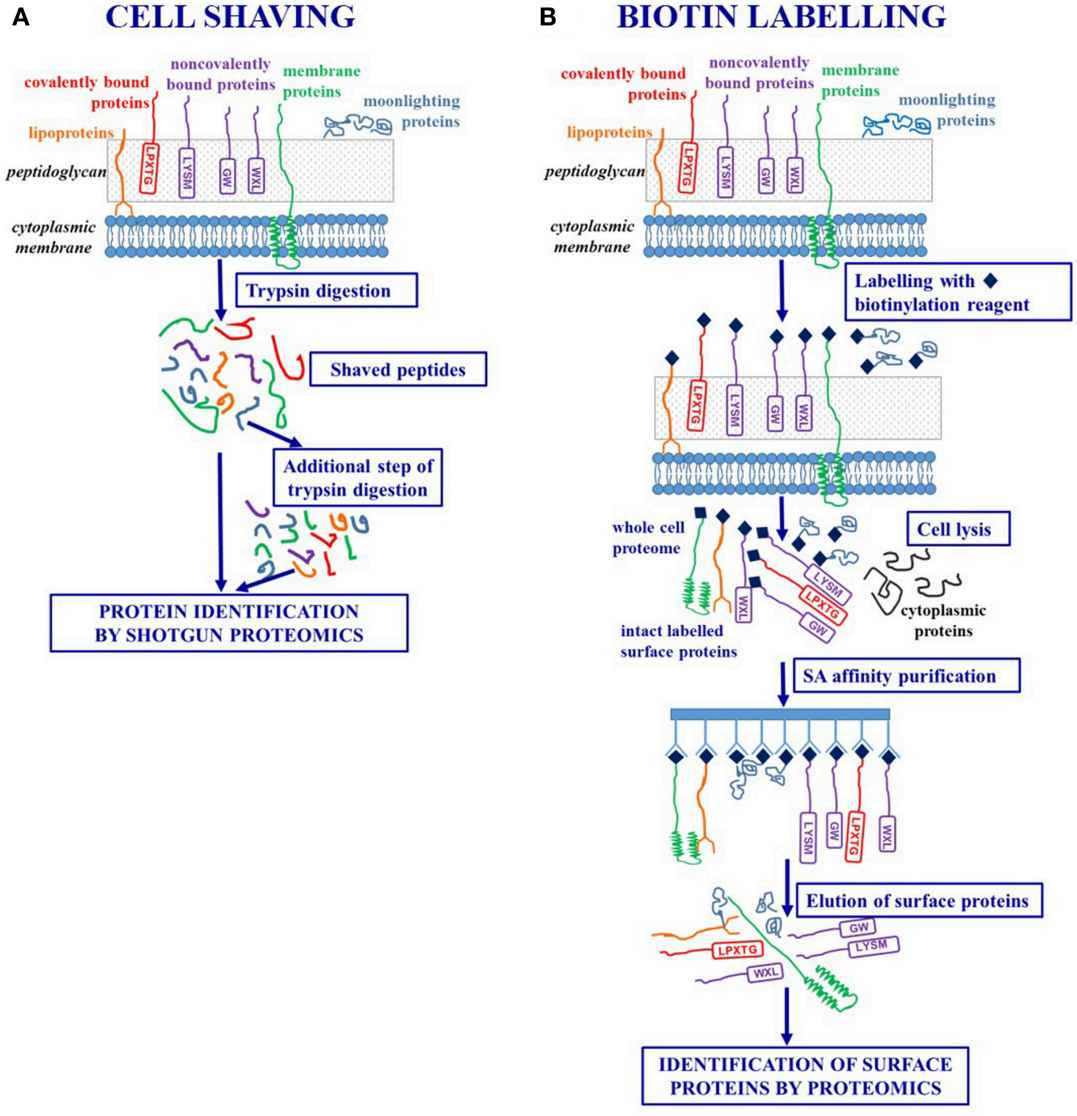
Workflows of *ad hoc* designed strategies for selectively targeting surface-exposed proteins. **(A)** Cell shaving strategies based on a limited proteolytic digestion step of intact bacterial cells in order to release surface exposed protein fragments. **(B)** Labeling strategies based on biotinylation of surface-exposed proteins of intact bacterial cells in order to achieve their purification by affinity chromatography.

The concept underlying this innovative strategy is that, regardless of anchoring mechanisms, surface exposed protein fragments can be selectively released through a limited proteolytic digestion step carried out on intact bacterial cells. The “shaved” peptides can be then separated from the whole cells by centrifugation and analyzed by shotgun proteomics thus achieving protein identification (39). Trypsin is the proteolytic enzyme more often used in these experiments, as lysine and arginine residues are widely represented in protein sequences and often easily accessible to the enzyme (Figures 1,2A).

In order to rule out osmotic shock and contamination of cytoplasmic proteins, bacterial cells are suspended in an isotonic buffer (obtained through the addition of sucrose or arabinose) and protease digestion is carried out for a short time (15–30 min) to preserve cell integrity. However, the limited proteolysis could produce high molecular weight protein fragments which are not amenable for LC-MS/MS analyses and a further extensive tryptic digestion step (18 hs) carried out on the shaved peptides has been included in optimized protocols (40, 41).

The cell shaving approach allows a comprehensive description of the bacterial proteosurfaceome and, as trypsin molecules could diffuse into the cell wall and interact with membrane and cell wall embedded proteins, a single experiment can provide the simultaneous identification of IMP, lipoproteins, LPXTG-proteins, non-covalently bound proteins, moonlighting proteins (40). Moreover, as the procedure selectively identifies peptides generated from surface-exposed domains, this methodology can contribute to refine the protein topology derived from bioinformatics by matching prediction and experimental data (39).

The method was conceived to identify potential targets of drugs and novel candidates for vaccines, and applied to study *Streptococcus pyogenes*, leading to the identification of 68 surface-associated proteins, one of which was validated in mice as a potential vaccine candidate (39, 42).

This protocol is particularly suitable for the analysis of Gram positive bacteria, due to their thick and rigid cell wall that is less prone to lysis during treatments, and comprehensive proteomic studies have been performed on several probiotics including *Lactococcus lactis* (43), *Bifidobacterium animalis* (44), *Lactobacillus rhamnosus* (45), *P. freudenreichii* (46, 47), and food pathogens like *Staphylococcus aureus* (48–50) and *Listeria monocytogenes* (51, 52). On the other hand, the cell shaving method has been applied for the analysis of surface proteins of Gram negative bacteria such as *Escherichia coli* (53) and *Salmonella enterica* (54), even if their cell envelope can be more easily damaged thus causing cytoplasmic protein contamination.

### Labeling of Surface-Exposed Proteins

These approaches rely on labeling strategies that specifically target surface proteins of intact bacterial cells. A first approach is based on the labeling of surface-exposed proteins with fluorescent CyDye reagents. After the labeling step, a complete cell lysis is carried out and the proteins are separated by 2-DE. Labeled surface proteins are highlighted by fluorescence imaging and identified using mass spectrometry (46, 55) (Figure 1).

A more sophisticated approach uses *ad hoc* designed biotin containing reagents to target surface proteins and suitably exploits the highly specific and stable non-covalent interaction between avidin [or the bacterial protein streptavidin (SA)] and biotin (56). As the biotinylation reagents can penetrate the peptidoglycan structure, proteins that are buried within the cell wall can be labeled. After a cell lysis step, labeled surface proteins are separated from non-labeled cytoplasmic proteins by SA affinity chromatography. The intact surface proteins thus purified could be identified using both classical proteomic approaches (including 2-DE) and Ge-LC-MS/MS or shotgun proteomics (27, 57–59) (Figures 1,2B).

The chemical features of the labeling reagent have a crucial role in this protocol. The biotin moiety is at the basis of the affinity purification step and several spacers can be linked to the valeric acid carboxylic group of biotin and carry functional groups (such as N-hydroxysuccinimide, NHS) that covalently link to primary amino groups of proteins (ε-amino group of lysine residues and N-terminal α-amine group) (60).

The elution of biotinylated proteins from SA-coated resin could be severely hampered by the high stability of the biotin-SA complex. To overcome this problem, cleavable spacers that include a disulphide bridge in their structure have been introduced thus allowing to easily and completely elute the biotinylated proteins by using reducing reagents such as dithiotreitol. Sulpho-NHS-SS-biotin is nowadays the most commonly used reagent in proteomic studies. The negative charge of sulphonate group present on the NHS ring makes this molecule water soluble and unable to cross the cell membrane thus drastically reducing the contamination of cytoplasmic proteins (60).

This method is suitable to study the proteosurfaceome of both Gram-positive and Gram-negative bacteria but, unfortunately, up to now, it has been mainly applied to analyze the surface proteins of pathogens such as *S. aureus* (27, 57, 61), *L. monocytogenes* (52), *E. coli* (62). We can foresee that in the near future, this strategy will be also applied to the characterization of the proteosurfaceome of probiotics thus contributing to the advancement of knowledge in this field.

## Proteomics for Studying Surface-Exposed Proteins In Probiotics

The high biological relevance of surface-exposed proteins in the dynamic crosstalk between bacteria and their environment prompted the design of dedicated proteomic strategies useful for investigating the molecular mechanisms of adaptation to GIT environment, adhesion, colonization, and immunomodulation, key features for the probiotic action (63–65). First studies on this topic, integrating proteomics and biochemical assays, confirmed the presence of moonlighting proteins on the surface of Lactobacilli and Bifidobacteria and their ability to bind extracellular matrix components (plasminogen, fibronectin, mucin) and adhere to GIT (28, 66–72). More recently, a comparative proteomic study performed on potentially probiotic strains of *Lactobacillus pentosus* led to identify moonlighting proteins in the cell-wall proteome and to correlate their abundance level to strain specific adhesion capacities to mucus (73).

Importantly, the adaptation to GIT can enhanced probiotic features as, for instance, bile stress can be a signal of gut entry for bacteria, which triggers the re-organization of the surface protein pattern resulting in improved adhesion ability in the new niche. In fact, the abundance of surface-exposed Clp proteins (ClpB, ClpE), chaperone (DnaK), and enzymes involved in carbohydrate metabolism was enhanced in *L. rhamnosus* GG after exposure to bile stress. The abundance of some of these enzymes increased in the proteosurfaceome but not in the proteome, thus suggesting a sort of protein relocalization triggered by bile (55). Changes in the expression profile of moonlighting proteins (in particular ribosomal proteins and glycolytic enzymes) promoted by bile were also observed in *B. animalis* subsp. *lactis* and *B. longum* (29, 74).

Several factors supplied with diets such as plant polyphenols (resveratrol, ferulic acid, etc.) and prebiotic carbohydrates (raffinose, fructooligosaccharides, etc.) could influence the surface protein profile of probiotics. These molecules improved the adhesive capabilities to mucin and HT-29 cells of *L. acidophilus* NCFM probably by modulating biosynthesis and/or secretion of moonlighting proteins (75–77).

Proteomics also contributed to assess that the surface protein pattern is strain specific. In fact, a trypsin shaving approach applied to compare the proteosurfaceomes of *L. rhamnosus* GG and the closely related dairy strain Lc705, led to the identification of 102 and 198 proteins anchored to the surface trough different mechanisms (IMP, LPXTG proteins, lipoproteins, C-term and N-Term anchored proteins and moonlighting proteins). Strain specific differences were mainly associated to moonlighting proteins and could be related to the adaptation to their ecological niches and probiotic functions (response to bile, hydrolysis of casein, immunostimulation, pathogen exclusion) (45). In addition, SpaC and SpaA proteins, involved in the assembly of pilus structures, were identified only in the proteome of GG and related to adhesion properties and prolonged residence in the GIT of this strain compared to Lc705 (78).

Similarly, a pilin-like protein was identified in a *L. lactis* strain able to adhere to Caco-2 cells and the synthesis of pili was confirmed by immunoblotting detection and electron and atomic force microscopy observations (43). Several proteins potentially involved in adhesion, including the pilus structure proteins FimA and FimB, were also identified in a *B. animalis* ssp *lactis* strain (44).

S-layer proteins (SLPs) and S-layer associated proteins (SLAPs) have a key role in GIT adaptation and immunomodulation processes, as highlighted by functional studies integrated by proteomics. First studies performed on *L. acidophilus* NCFM, a S-layer forming microorganism, showed the involvement of the main SLP (SlpA) in adhesion to Caco2 cells (79) and dendritic cells (DCs) immunomodulation (80). Recently, using an optimized extraction protocol, 37 SLAPs of L*. acidophilus* NCFM were identified and most of them were predicted to be extracellular proteins while only four proteins were potentially moonlighting proteins. The protein SLAP LBA1029 contributed to a pro-inflammatory response in murine DCs (inducing the expression of TNF-α), thus indicating that SLAPs may impart immunological properties to microbes (33). The surface-exposed proteins of *L. acidophilus* NCFM were also characterized by Celebioglu and Svensson (35), leading to the identification of a higher number of moonlighting proteins. In addition, a quantitative proteomic study performed using more sophisticated mass spectrometric approaches, defined a detailed catalog of the *L. acidophilus* NCFM surface proteins containing 276 SLAPs and demonstrated that the cell surface proteome was modulated by growth phase. This feature could be exploited to optimize probiotic actions and enhance their delivery, persistence, and general efficacy (36).

The presence or absence of the S-layer has a clear impact on the composition and complexity of proteosurfaceome of different lactobacilli. In fact, a proteomic study led to identify numerous SLAPs in proteosurfaceome of S-layer-forming strains of *L. acidophilus, L. helveticus, L. crispatus, L. amylovorus, and L. gallinarum*. On the other hand, the few proteins isolated with LiCl treatment of the non-S-layer forming strains of *L. delbrueckii subsp. bulgaricus and L. casei* were mostly intracellular proteins, likely presented extracellularly due to cell lysis in stationary phase. These findings also confirmed the role of SLAPs as integral components of S-layer (34).

Recently surface proteins from *P. freudenreichii*, a S-layer forming bacterium, have been characterized by using three complementary proteomic methods (guanidine hydrochloride extraction, cell shaving and fluorescent labeling with CyDye coupled to mass spectrometric analyses) and their ability in enhancing the production of the anti-inflammatory cytokines IL6 and IL10 by human immune cells was assessed (46). Furthermore, a study combining comparative genomics, transcriptomics and surface proteomics, coupled with gene inactivation, was performed on 23 strains of *P. freudenreichii* with different anti-inflammatory potential. Results evidenced the involvement of SlpB and SlpE in the release of IL10 by human immune cells and demonstrated that different combinations of surface and cytoplasmic proteins, depending on the strain, exerted a pleiotropic effect on the anti-inflammatory properties (47). Finally, do Carmo et al. demonstrated a direct role of SlpB in the adhesion to HT-29 cells and reported that the inactivation of *slpB* gene caused profound modifications in whole cell and surface proteomes as well as bacterial stress tolerance (81, 82).

The reported achievements clearly assess the ability of proteomics in investigating the structural features of different classes of surface proteins and its role in elucidating the mechanisms of bacteria/host interaction.

## Concluding Remarks

In the past few years, proteomics proved to be the method of choice to investigate surface architecture of bacteria cells, contributing to define protein location and topology, and to deal with the extremely dynamic and constantly renewing nature of proteosurfaceome that is deeply affected by the environment. The exploitation of technological innovations in proteomics and mass spectrometry has improved the knowledge in the probiotics field. This review displays the essential role of proteomics in the elucidation of the molecular mechanisms of the probiotic action and the identification of key actors of the probiotics/host molecular dialogue, thus potentially providing new tools for selecting strains with specific healthy promoting functions. Future research perspectives should include the analysis of post-translational modifications of surface proteins (glycoproteomics, phosphoproteomics, etc.), and the investigation of their effects in the interaction with host epithelial cells.

## Author Contributions

All authors listed have made a substantial, direct and intellectual contribution to the work, and approved it for publication.

### Conflict of Interest Statement

The authors declare that the research was conducted in the absence of any commercial or financial relationships that could be construed as a potential conflict of interest.
